# Creation of TGMS Lines of Waxy Rice with Elite Physicochemical Properties of Starch via Waxy Gene Editing

**DOI:** 10.3390/foods14203530

**Published:** 2025-10-16

**Authors:** Jun Zhu, Zhenchao Wang, Ruipeng Zhao, Weiyi Li, Tanghuang Gan, Jiaxin Wan, Haoliang Sun, Ying Liu, Min Wei, Hongyan Xu, Tingting Luo, Yonghuan Hua, Shuangcheng Li, Yuhao Fu, Ping Li

**Affiliations:** 1 Rice Research Institute, Sichuan Agricultural University, Chengdu 611130, China; zhujun987@126.com (J.Z.); zcwang2021@163.com (Z.W.); 18080170760@163.com (R.Z.); 13340961577@163.com (W.L.); g1986469541@163.com (T.G.); 18798538240@163.com (J.W.); xsxsmmczdyx@163.com (H.S.); liuy971220@163.com (Y.L.); 18384376465@163.com (M.W.); 15326676004@163.com (H.X.); lttjoy@163.com (T.L.); 18093195686@163.com (Y.H.); lisc926105@163.com (S.L.); 2 Yibin Academy of Agricultural Sciences, Yibin 644000, China; 3 State Key Laboratory of Crop Gene Exploration and Utilization in Southwest China, Sichuan Agricultural University, Chengdu 611130, China

**Keywords:** waxy rice starch, hydrocolloidal properties, waxy gene, gel properties, CRISPR/Cas9

## Abstract

Waxy rice starch (WRS), characterized by low amylose content, high viscosity, and strong gel-forming ability, is highly valued in food and industrial applications. Temperature-sensitive genic male-sterile (TGMS) lines exhibit complete male sterility under low-temperature conditions, a trait widely exploited in hybrid rice breeding. Here, we generated an elite waxy TGMS line, 520S, via CRISPR/Cas9-mediated editing of the Waxy (*Wx*) gene. The *wx* mutants displayed robust male sterility, desirable glutinous traits, and favorable physicochemical properties, including gelatinization temperature, gel consistency, paste viscosity, and amylopectin fine structure. Fertility assays confirmed temperature-sensitive pollen sterility consistent with wild-type responses, and T2 generation mutants were transgene-free with stable inheritance of the waxy phenotype. Notably, *wx* starch maintained gel stability over 48 h, demonstrating superior hydrocolloidal properties and translucency compared with wild-type and commercial WRS. 520S*wx1* retained gelatinization temperature and amylopectin structure comparable to wild type, highlighting the potential of CRISPR/Cas9-mediated mutagenesis to enhance waxy rice yield while preserving starch quality. These findings establish an efficient strategy to improve both production and functional performance of WRS for industrial and food applications.

## 1. Introduction

Waxy rice holds significant cultural and culinary importance across several Asian countries [1,2,3]. Unlike conventional rice varieties, waxy rice contains extremely low or negligible amylose content (AC), which is a key determinant of its distinctive physicochemical properties. Rice is typically classified into five categories based on AC: waxy (≤2%), very low (2.1–9.0%), low (9.1–20.0%), medium (20.1–25.0%), and high (≥25.1%) [4]. The absence of amylose in waxy rice and its starch (WRS) imparts unique textural and gel-forming characteristics, facilitating widespread applications in food, industry, and medicine [5,6].

Starch, the major component of rice endosperm, comprises two glucans: amylose and amylopectin. Amylose is largely linear, whereas amylopectin is highly branched, and the relative proportions of these polymers govern rice cooking, processing, and sensory qualities [7,8,9,10]. Gelatinization temperature (GT) is influenced by amylopectin fine structure, whereas AC affects gel formation and water solubility [11]. Desirable WRS gels typically display smoothness, glossiness, and minimal stickiness [12].

Recently, temperature-sensitive genic male-sterile (TGMS) lines have emerged as pivotal tools in rice breeding, particularly in two-line hybrid systems that combine superior varieties [13,14,15,16]. Enhancing the genetic resources of TGMS waxy germplasm and accelerating the development of hybrid waxy rice are thus priorities for breeders. TGMS lines not only enable the creation of high-yielding, high-quality hybrids, often increasing yields by ≥10%, but also facilitate the improvement of conventional varieties with favorable agronomic traits [17]. Notably, CRISPR/Cas9-mediated mutagenesis has been successfully applied to generate new waxy TGMS lines and two-line hybrids [18,19]. Our recent work further demonstrates that targeted editing of the *Wx* gene in TGMS lines with low initial AC can produce *wx* mutants with reduced AC while retaining GT and amylopectin fine structure [11,20]. This strategy offers an efficient route to high-yield WRS with tailored starch properties, providing a valuable framework for both breeding and industrial applications.

In this study, we established a simple and efficient breeding strategy for indica waxy rice using the CRISPR/Cas9 system (Baige, Jiang Su, China). The resulting waxy TGMS line, 520S*wx1*, exhibits considerable potential for cultivation in southwest China. The 520S wild-type line combines favorable traits, including low amylose content, optimal plant architecture, desirable physicochemical properties, and high yield potential. Targeted CRISPR/Cas9 editing of the *Wx* gene in 520S thus provides a promising avenue for expanding high-quality waxy rice cultivation and enhancing starch production.

## 2. Materials and Methods

### 2.1. Plant Materials and Growth Conditions

The indica rice cultivar ‘520S’, a TGMS variety known for its heavy panicles and tall stems, was used. A CRISPR/Cas9 vector targeting the *Wx* gene was constructed as previously described [21]. Specifically, the designed sgRNA was cloned into the pCAMBIA1300-Cas9 backbone and verified by sequencing. Mature embryo-derived calli were induced on 2,4-D-containing medium and transformed via Agrobacterium tumefaciens strain EHA105. Transgenic calli were selected on antibiotic-containing medium, regenerated into plants, and confirmed by PCR and Sanger sequencing for the presence of Cas9 and target gene mutations [22]. Primer sequences are provided in Supplementary Table S1. Transgenic lines were grown in a confined field trial under natural conditions in Chengdu, China, with all necessary biosafety permits and regulatory compliance. Appropriate isolation measures were applied to prevent gene flow. T1 and T2 seeds were harvested and subjected to the same germination and drying procedures. Waxy rice starch, used as a control, was purchased from a local supermarket in Chengdu.

### 2.2. Phenotype and Genotype Assays

Agronomic traits (plant height, effective panicle number, grain number per panicle) were measured in T2 plants. Thousand-grain weight, grain length, and width were determined using an SC-A grain analysis system (Wseen Co., Zhengjiang, China). Wild-type 520S plants were used as controls for comparison of agronomic traits and genotypic analyses. Genomic DNA was extracted from the young leaves of T0, T1, and T2 plants as described previously [23]. The polymerase chain reaction (PCR) amplification was performed using gene-specific primers. The PCR products were first checked by agarose gel electrophoresis, purified using a commercial gel extraction kit, and then sequenced by the Sanger method to confirm target site mutations in the *Wx* gene. The PCR amplification conditions were as follows: 94 °C for 2 min; 35 cycles of 94 °C (30 s), 56 °C (30 s), 72 °C (30 s); final extension 72 °C for 5 min. The sequences of detection primers are listed in Table S1.

### 2.3. Grain Morphology and Endosperm Starch Staining

Dehulled grains of the wild-type and 520S*wx1* lines were visually inspected for external morphology. For iodine staining, grains were transversely sectioned at approximately 1/3 of the length from the embryo end to expose the endosperm. A 1% iodine solution was applied to the cut surface, the low-amylose waxy rice endosperm stained reddish-brown or remained colorless after 1 min.

### 2.4. Pollen Viability Assay

During the flowering stage, five unopened panicles were collected from each rice line. Unopened spikelets were sampled from the upper, middle, and lower parts of each panicle. Anthers were carefully removed and crushed on a glass slide to release pollen grains. Pollen viability was assessed by staining with an I_2_-KI solution and examined under a light microscope. Pollen grains containing starch stained dark brown to black, indicating fertility, while sterile pollen grains without starch stained light yellow.

### 2.5. Total Protein and Amylose Content Determination

Seeds from homozygous transgenic plants were harvested at maturity and air-dried. Total protein content was measured using the Kjeldahl method [24]. For amylose content determination, a modified iodine colorimetric method was employed. 0.1 g of defatted rice starch was weighed into a 100 mL volumetric flask. After adding 1 mL of 95% ethanol and 9 mL of 1 M NaOH, the mixture was heated in a boiling water bath for 10 min to gelatinize the starch. The solution was then cooled and diluted to 100 mL with ddH_2_O. A 5 mL aliquot of this solution was pipetted into a 100 mL volumetric flask, followed by the addition of 1 mL of 1 M acetic acid and 1 mL of iodine reagent. The volume was brought to 100 mL with ddH_2_O, mixed thoroughly, and left to stand for 10 min for color development. The absorbance was measured at 620 nm using a UV-Vis spectrophotometer (Thermo Fisher Scientific, Waltham, MA, USA). A blank solution, prepared in the same way but without rice starch, was used as a reference. The amylose content was calculated based on a standard curve of potato amylose.

### 2.6. Scanning Electron Microscopy

Rice grains were dried in an oven at 42 °C for at least 2 days. The dried grains were manually fractured to expose the cross-section, which was then mounted on a sample stub and sputter-coated with a thin layer of gold-palladium. The morphology of the endosperm and starch granules was observed using a Hitachi S-4800 scanning electron microscope (Hitachi High-Technologies Corporation, Tokyo, Japan) at 50 and 1000 power [25].

### 2.7. Gel Consistency

Gel consistency was determined according to the method described by [26]. A 100 mg sample of rice starch was accurately weighed into a 13 × 150 mm test tube. After adding 200 μL of a 0.025% bromophenol blue solution in 95% ethanol, the mixture was vortexed thoroughly. A 2 mL of 0.20 M NaOH solution was added, and the test tube was immediately placed in a boiling water bath for 8 min, with a glass bead placed on top to minimize evaporation. The test tube was then cooled in a 0 °C ice-water bath for 20 min. The length of the gel column was measured on a scale with the test tube laid horizontally after 1, 24, and 48 h.

### 2.8. Pasting Properties by Rapid Visco Analyzer

The pasting properties of rice starch were analyzed using a Rapid Visco Analyzer (RVA, RVA-4500, Perten, Stockholm, Sweden). A 3.0 g sample of rice starch (at 12% moisture content) was weighed into an RVA canister, and 25 mL of dH_2_O was added. The slurry was rapidly stirred to disperse the sample. The RVA temperature profile was programmed as follows: The sample was held at 50 °C for 1 min, then heated to 95 °C at a rate of 12 °C/min, held at 95 °C for 2.5 min, cooled to 50 °C at 12 °C/min, and finally held at 50 °C for 2 min. The total run time was 12.5 min. The stirring speed was maintained at 960 rpm for the first 10 s and then reduced to 160 rpm for the remainder of the analysis. The following pasting parameters were recorded from the resulting viscoamylogram: peak viscosity (PKV), trough viscosity (TRV), final viscosity (FV), breakdown viscosity (BDV = PKV − TRV), setback viscosity (SBV = FV − PKV), and pasting temperature (PaT).

### 2.9. Thermal Properties by Differential Scanning Calorimetry

The thermal properties of the starch samples were analyzed using a Differential Scanning Calorimeter (DSC Q2000, TA Instruments, New Castle, DE, USA). A 2 mg sample was weighed into an aluminum crucible, and 6 μL of ddH_2_O was added to obtain a solid-to-liquid ratio of 1:3 (*w*/*w*). The crucible was hermetically sealed and allowed to equilibrate for 2 h at room temperature. The crucible was then heated from 30 °C to 110 °C at a heating rate of 10 °C/min, using an empty crucible as a reference. The gelatinization onset temperature (*T_o_*), peak temperature (*T_p_*), end temperature (*T_c_*), and enthalpy of gelatinization (Δ*H*) were calculated from the resulting thermograms.

### 2.10. X-Ray Diffraction (XRD)

The crystalline structure of the rice starch samples was determined using an X-ray diffractometer (Rigaku D/maxA, Tokyo, Japan). The powdered sample was packed into a glass holder and gently pressed to create a flat surface. The XRD patterns were recorded at a voltage of 40 kV and a current of 40 mA, with a scanning range of 2° to 90° (2θ) at a scan rate of 2°/min. The relative crystallinity was calculated using the area of the crystalline peaks relative to the total area of the diffraction profile [27]. The resulting data were plotted using Origin 2021 software.

### 2.11. Fourier Transform Infrared Spectroscopy

FTIR spectroscopy was performed using a Thermo Fisher Scientific Nicolet iS5 spectrometer (Waltham, MA, USA) in attenuated total reflection (ATR) mode. Spectra were acquired over the wavenumber range of 500–4000 cm^−1^. The resulting ATR spectra were converted to absorption spectra using Omnic 8.2 software. To evaluate the molecular order of the starch, the intensities at 1022 cm^−1^ and 1047 cm^−1^ were used to calculate the ratio of the crystalline to amorphous regions.

### 2.12. Amylopectin Chain-Length Distribution

The chain-length distribution of amylopectin was determined by HPAEC-PAD (Dionex ICS-5000, Waltham, MA, USA). A 5 mg starch sample was gelatinized in 5 mL of ddH2O in a boiling water bath for 60 min. The solution was then enzymatically debranched by adding isoamylase (3.5 μL), sodium acetate buffer (125 μL, 600 mM, pH 4.4), and sodium azide (25 μL, 2%) and incubating at 38 °C for 24 h. The resulting debranched glucan chains were reduced by adding 375 μL of a 2% sodium borohydride solution and incubating for 24 h. The reaction was terminated by adding acetic acid. The reduced samples were filtered through a 0.45 μm syringe filter and injected into the HPAEC-PAD system. The separation was achieved on a CarboPac PA-100 column using a gradient elution with 100 mM NaOH (Eluent A) and 100 mM NaOH with 1 M NaAc (Eluent B). The flow rate was 0.4 mL/min, and the column temperature was maintained at 30 °C.

### 2.13. Statistical Analysis

All experiments were performed in at least three biological replicates. Data are presented as the mean ± standard deviation (SD). Statistical significance was determined using Student’s *t*-test or one-way analysis of variance (ANOVA), and differences were considered significant at *p* < 0.05 (* or lowercase letters) and *p* < 0.01 (** or uppercase letters). All graphs were prepared using OriginPro 2021 software.

## 3. Results and Discussion

### 3.1. Generation of a New Wx TGMS Line

**Figure 1:**
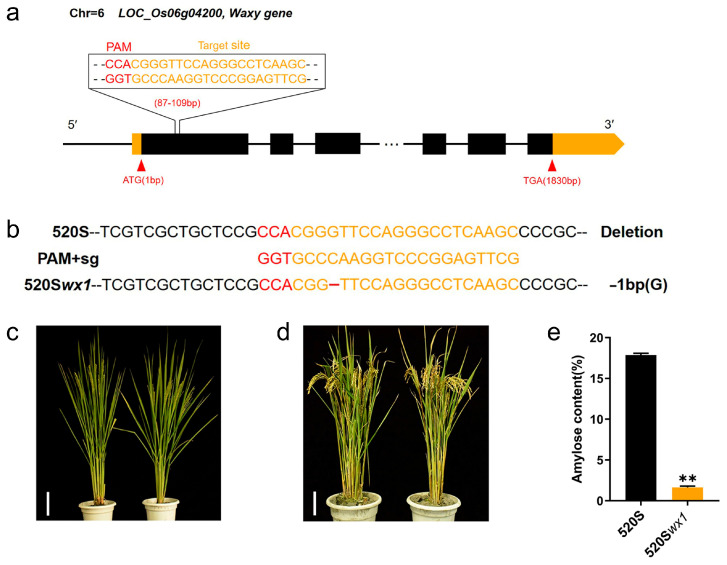
CRISPR/Cas9-targeted mutagenesis of the Waxy gene in TGMS lines. (**a**) Schematic diagram of the targeted site in the Waxy gene (*LOC_Os06g04200*). Red arrows indicate the start codon and stop codon. The numbers in brackets indicate the distance to the start codon (ATG). The sequence of the targeted site is shown with the protospacer adjacent motif (PAM) sequences labeled in red color. (**b**) Examples of mutations at the Waxy locus in CRISPR-waxy T0 generation plants of 520S and 520S*wx1*. The targeted sequence is highlighted in orange and the PAM sequences are in red. The targeted sequence is highlighted in red and the PAM sequences are in blue. Mutations are marked in red color. (**c**,**d**) Phenotypes of CRISPR-waxy mutants and their corresponding WTs. (**e**) AC of *wx* mutants and their corresponding WTs (**, *p* < 0.01).

Using the CRISPR/Cas9 system, we successfully generated a new waxy TGMS line by introducing a loss-of-function mutation in the *Wx* gene (Figure 1a,b). Our editing approach achieved high editing efficiencies (92.3% in T0 plants) with low off-target effects, as demonstrated by the target sequence analysis (Table S2) [19,28]. From the edited lines, we carefully selected a single-base homozygous *wx* mutant, 520S*wx1*, to evaluate its major agronomic traits, AC, and fertility (Figure 1b). Notably, the major agronomic traits of the T2 generation *wx* mutants remained unchanged compared to the WT, including panicle traits, spikelet number per panicle, grain length (Figure 1c,d and Figure S1a). 520S*wx1* showed the lowest AC level (1.63%) compared with its corresponding WT (17.89%) (Figure 1e and Table 1). Importantly, 520S*wx1* exhibited the lowest AC among the *wx* mutants and its corresponding WT, confirming its status as a true waxy rice variant, and confirmed that its utilization in hybrid waxy rice breeding and the production of WRS [29,30].

We further examined the temperature sensitivity of fertility transformation in the mutants and observed that it was similar to the WT control. When the temperature exceeded 24 °C, I_2_-KI staining of pollen from both *wx* mutants and WT turned red-brown, indicating infertility (Figure S1b). Conversely, when the temperature dropped below 24 °C, I_2_-KI staining of pollen from both *wx* mutants and WT appeared dark blue, indicating fertility (Figure S1b). We also had found that the mutants were “transgene-clean” plants of T2 generation, and the waxy characteristics was genetic stability (Figure S2). Our results are consistent with previous findings and further clarify the effectiveness of this strategy [31,32,33].

### 3.2. Grain Quality

**Figure 2:**
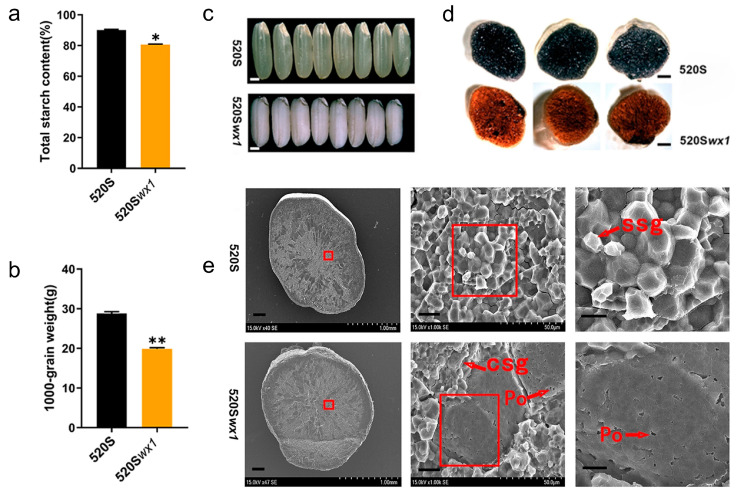
Total starch content, 1000-grain weight, Grain phenotypes and scanning electron micrographs of endosperms in mature seeds of *wx* mutants and their corresponding WTs. (**a**) Total starch content of *wx* mutants and WT (*, *p* < 0.05; **, *p* < 0.01). (**b**) 1000-grain weight of *wx* mutants and WT. (**c**) Grain phenotypes of *wx* mutants and WT. Upper row, endosperm phenotype of the WT seeds. Bottom row, endosperm phenotypes of the *wx* mutants. (**d**) Iodine-staining of endosperm in cross-sections of seeds of *wx* mutants and WT. Upper row, iodine-staining of endosperm in cross-sections of WT seeds. Bottom row, iodine-staining of endosperm in cross-sections of the *wx* mutants. (**e**) Scanning electron micrographs of endosperms. WT seeds exhibit tightly packed, polygonal single-starch granules (ssg), while *wx* mutants display irregular composite starch granules (csg) with visible pores (Po) distributed among the starch structure. The red frame indicates the scanning area.

The reduction in AC in the *wx* mutants led to a further decrease in total starch content (Figure 2a). Additionally, we observed a significant reduction in the 1000-grain weight of the *wx* mutants compared to the WT, a common phenomenon observed in various waxy rice breeding studies (Figure 2b) [28,34]. Morphologically, the mutant seeds displayed a distinctive “*wx*” appearance, characterized by a milky white and fully opaque texture, in stark contrast to the typical translucent appearance of “non-waxy” WT seeds (Figure 2c). Cross-sections of the *wx* mutant seeds exhibited a red-brown coloration when stained with an iodine solution, indicating a lower amylose/amylopectin ratio compared to the dark blue coloration observed in WT endosperms (Figure 2d). The observed similarities in grain cross-section and starch structure to those of naturally glutinous rice varieties further support that the *wx* mutant possesses the defining physicochemical characteristics of glutinous rice [35,36].

Based on the findings from scanning electron microscopy, notable disparities were observed in the starch structure of the grain cross-sections between the *wx* mutants and WT seeds. Specifically, the cross-section of the *wx* mutant revealed irregular composite starch granules (CSg) with a notable abundance of small pores (Po) distributed among the starch grains. Conversely, the cross-section of WT seeds exhibited numerous polygonal single-starch granules (SSg) (Figure 2e). This distinct starch morphology observed in the *wx* mutants is likely a contributing factor to the observed reduction in the 1000-grain weight when compared to the WT.

### 3.3. Cooking Quality

**Figure 3:**
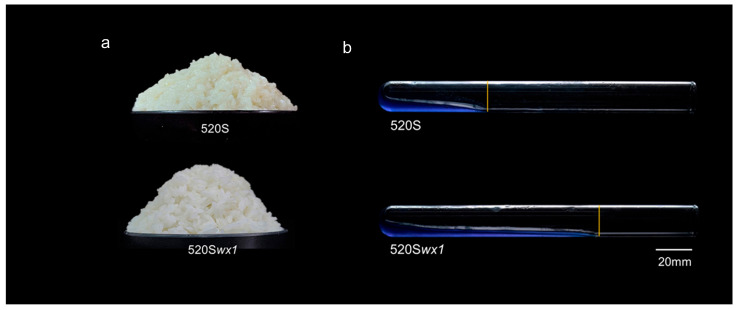
The cooking quality of rice. (**a**) The appearance of cooked waxy rice and their corresponding WTs. (**b**) Gel consistency of *wx* mutant and their corresponding WTs.

Improving the eating and cooking qualities through *Wx* gene editing is evident in our study. The *wx* mutant exhibited cooked rice grains that are non-sticky and maintain separation, a highly sought-after characteristic for consumers (Figure 3a). Examination of gel consistency revealed that the *wx* mutant displayed a softer gel compared to its WT counterpart (Figure 3b and Table 1). Analyzing flour gel consistency (GC) further supported our findings, indicating that the reduction in AC corresponded to a higher GC compared to the WT, in line with previous research [37,38]. These findings demonstrate that the novel waxy rice generated through *Wx* gene editing exhibits superior characteristics, including a softer gel texture, thereby enhancing the overall quality of the rice grains.

### 3.4. Gel Properties

**Figure 4:**
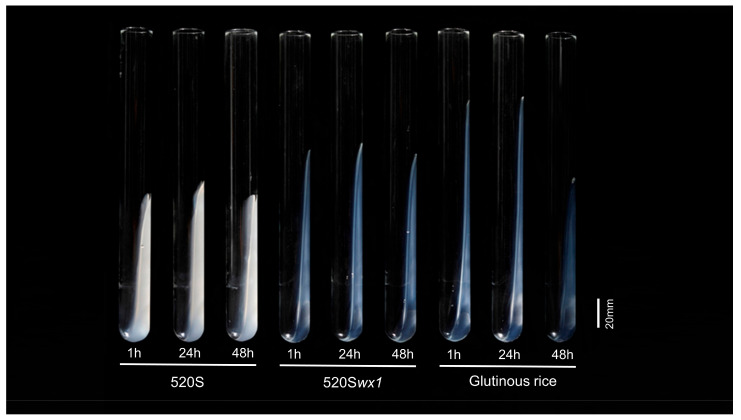
Starch Gel consistency of *wx* mutant starches and their corresponding WTs.

Starch gel analysis serves as an intuitive approach for evaluating the physicochemical properties of flours and starches, providing a convenient means to predict diverse starch characteristics [39]. Our examination of starch GC yielded compelling results, indicating that the reduction in AC corresponded to a higher GC compared to the WT, which aligns with previous findings (Figure 4 and Table 1). Similarly, the starch hydrocolloid of the *wx* mutant exhibited a significant increase compared to the WT, indicating that starch gelation properties play a substantial role in determining the gel characteristics of rice flour. On the other hand, it was easily observable from the aqueous colloidal state that the mutant displayed superior clarity and glossiness compared to the WT. Straight-chain starch, upon gelatinization, remains insoluble in water, resulting in opaque starch gels [40,41]. Lower AC correlates with increased starch solubility and transparency, while the branched structure of starch similarly impacts the transparency of the hydrocolloid [12]. Our experiments notably enhanced the transparency and glossiness of rice starch hydrocolloids, aligning with the desired attributes of high-quality waxy rice starch. Furthermore, extending the analysis over time intervals of 24 and 48 h revealed consistent stability in the hydrocolloids of both the WT and the mutant, with no significant changes observed, whereas commercially available WRS displayed poor stability (Figure 4 and Table 1). These results underscore the outstanding physicochemical properties of WRS produced through this method, thereby enhancing the potential applications of WRS.

### 3.5. Pasting and Thermal Properties of Starches

**Figure 5:**
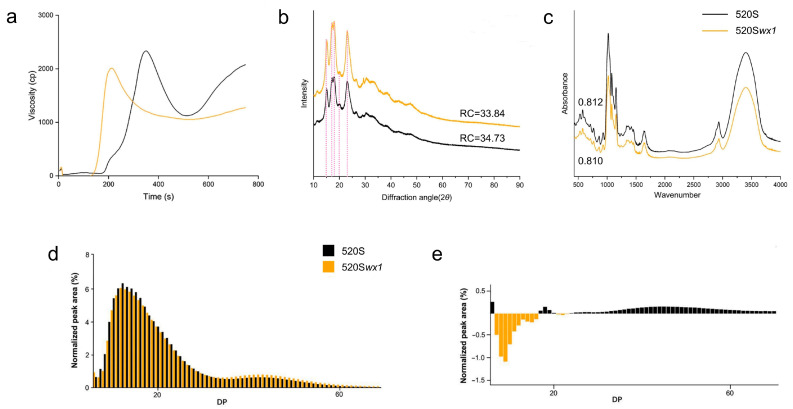
RVA profiles, X-ray diffraction patterns, Fourier transforms infrared spectroscopy, HPAEC-PAD chromatograms, and Comparison of percentage values of amylopectin chain length. (**a**) RVA profiles of *wx* mutant starches and WT. 15°, 17°, 18°, 20°, and 23°are marked with a pink dotted line. (**b**) X-ray diffraction patterns of *wx* mutant and their corresponding WTs. The numbers of (**b**) at right are the relative crystallinity. (**c**) Fourier transforms infrared spectroscopy of *wx* mutant and their corresponding WTs. The numbers of (**c**) at light are the ratio of 1047 cm^−1^ to 1022 cm^−1^. (**d**) The difference in the chain-length distribution of *wx* mutant starches and WTs. (**e**) Comparison of percentage values of amylopectin chain length between *wx* mutants and WT.

RVA (Rapid Visco Analysis) profiles serve as a useful tool for simulating the pasting process of starch. In the *wx* mutants, the reduction in AC led to lower pasting properties compared to their corresponding WT (Figure 5a). Specifically, the CPV demonstrated a significant decrease, which can be attributed to the reduction in AC [42,43]. Waxy starch typically exhibits negative values in RVA profiles, indicating a tendency towards stronger retrogradation of starch pastes. Furthermore, our study unveiled a correlation between higher AC and prolonged peak times in the RVA profiles. Notably, the RVA results highlighted that the *wx* mutant showcased softer gel characteristics.

The thermal parameters of starch analyzed by DSC can be classified into four data points: *To*, *Tp*, *Tc*, and Δ*H*. Tp generally represents the GT. In our study, the starch of *wx* mutants exhibited a GT similar to that of the WT, while its ΔH showed a significant increase (Table 2). Previous studies have suggested that the crystallinity of waxy or *wx* mutant starch is higher compared to normal one, indicating a higher energy requirement for gelatinization, resulting in the observed high Δ*H* [44,45]. Moreover, we observed a discrepancy between the pasting temperature and GT, leading us to conclude that pasting temperature in RVA analysis cannot precisely represent the GT of the starch samples.

Indeed, the evidence consistently supports the notion that GT in *wx* mutants remains unchanged following *Wx* gene editing. This phenomenon has been observed in various studies, including the present investigation. Consequently, the findings suggest that it is feasible to produce WRS with the desired GT and achieve high yield through *Wx* gene editing. This promising outcome holds significant potential for advancing the production of WRS with specific characteristics [11,46].

### 3.6. Crystalline Structure and FTIR Spectroscopy

All rice starches exhibited an A-type pattern, characterized by a doublet at 17° and 18°, along with individual peaks at 15°, 20°, and 23° [47]. Interestingly, our findings suggest no notable distinction in crystallinity between the mutant and the WT, diverging from prior research outcomes (Figure 5b). This discrepancy implies that this material differs from other glutinous rice starch materials generated through *Wx* gene editing, potentially offering unique applications [48].

Absorption peaks at 1047 cm^−1^ and 1022 cm^−1^ signify crystallization and amorphous areas in rice starch granules, respectively [49]. The ratio of intensities at 1047 cm^−1^ and 1022 cm^−1^ reflects the ratio of crystalline to amorphous domains. Both the mutant and WT displayed ratios of 1047/1022 as 0.812 and 0.810, respectively, with no statistically significant difference between them, corroborating the XRD results (Figure 5c). These results indicate that the starch crystallinity of the mutant does not change significantly compared with the wild type after gene editing, providing useful data for the targeted production of specific waxy rice varieties.

### 3.7. Fine Structure of Amylopectin

The isoamylase debranched *wx* mutant starches were subjected to analysis using HPAEC-PAD chromatograms (Figure 5d). The results revealed significant alterations in the chain length distributions of *wx* mutants compared to the WT (Figure 5e). It is worth noting that amylopectin fine structure is linked to GT, which can be classified into two types based on ACR (amylopectin chains ratio) values: LGT (low) and HGT (high) [20,50,51,52]. However, an essential finding from our study was that despite the changes in chain length distributions, the amylopectin fine structure type remained consistent with the WT in *wx* mutants (Table 2). As a result, we can confidently conclude that GT will not be altered between the *wx* mutants and WT. This conclusion was further substantiated by our DSC analysis, providing strong evidence for the stability of GT in the *wx* mutants again.

### 3.8. Potential Application

Building on our molecular work, the successful generation of the elite waxy TGMS line 520S*wx1* demonstrates not only the feasibility of precise GT editing in TGMS backgrounds but also provides a concrete strategy for breeding high-quality hybrid waxy rice. The observed maintenance of key agronomic traits in 520S*wx1* suggests that CRISPR/Cas9-mediated editing of the *Wx* gene can be effectively integrated into elite breeding lines without compromising yield or plant type. Moreover, the superior transparency, glossiness, and non-adhesive properties of the *wx* starch, together with its enhanced stability during storage and processing, highlight its potential for broad applications in food processing, especially in products sensitive to starch aging [18,53]. Collectively, these findings suggest that targeted molecular modifications can be leveraged to develop hybrid waxy rice varieties with predictable starch functionality, providing breeders with actionable guidance for both trait selection and product-oriented quality improvement.

Furthermore, CRISPR/Ca9-mediated editing of the *Wx* gene in elite rice varieties has been shown to improve yield by 10–15% compared to normal waxy rice varieties [20]. Additionally, the two-line hybrid approach has demonstrated a yield increase of at least 10% in rice breeding [15,54]. Capitalizing on the TGMS-based strategy, we successfully developed new hybrid rice varieties using 520S, resulting in a remarkable yield increase of 10–20% compared to normal varieties in the western region of China. In conclusion, our study provides an accessible and efficient method to produce waxy rice or WRS with high yield, promising advancements in quality waxy rice breeding and its starch manufacturing.

Admittedly, the emergence of the CRISPR/Cas system has brought unprecedented advantages to targeted rice breeding. However, concerns regarding potential transgenic risks and strict government regulations have hindered its broad application. Gene editing based on the CRISPR/Cas system has been demonstrated not to introduce foreign genetic fragments, but the newly created alleles or mutants may not have corresponding natural variants, which has raised widespread debate. Nevertheless, several commercial rice varieties have already been developed using CRISPR/Cas to reproduce naturally occurring mutations. Such lines, including the *wx* mutant, pose no biosafety risks and can be safely promoted and applied under appropriate regulatory frameworks [55].

## 4. Conclusions

In conclusion, we have successfully generated an elite TGMS waxy rice line, 520S*wx1*, through CRISPR/Cas9-mediated *Wx* gene editing. 520S*wx1* retains the favorable agronomic traits of its wild-type progenitor, including optimal plant architecture, high yield potential, and superior gel properties. Its starch maintains gel strength for over 48 h, while gelatinization temperature and amylopectin fine structure remain unaltered, demonstrating that targeted gene editing can enhance waxy rice yield without compromising starch quality. Notably, the crystallinity and gel characteristics of 520S*wx1* closely resemble those of the wild type, contrasting with previous reports and highlighting the need for further mechanistic studies. These findings establish a robust strategy for producing high-quality waxy rice with tailored functional properties, offering promising applications in both hybrid rice breeding and industrial starch production.

## Figures and Tables

**Table 1:** Amylose content, Rice noodles gel consistency, starch gel length of *wx* mutant and corresponding WT.

Cultivar	AC(%)	GC of Flour (mm)	GC of Starch (mm)		
			1 h	24 h	48 h
520S	17.89 ± 0.12 ^a^	59.34 ± 0.42 ^a^	83.24 ± 0.16 ^a^	86.12 ± 0.37 ^a^	83.09 ± 0.12 ^a^
520S*wx1*	1.63 ± 0.34 ^b^	102.56 ± 0.31 ^b^	104.35 ± 0.25 ^b^	106.31 ± 0.19 ^b^	103.52 ± 0.34 ^b^

“Gel Consistency” is abbreviated as “GC”. Values in the same column with the different letter is significantly (*p* < 0.05).

**Table 2:** The gelatinization temperature and distribution of amylopectin chain-length of *wx* mutant starch and corresponding its WT.

Cultivar	*T_o_* (℃)	*T_p_* (℃)	*T_c_* (℃)	Δ*H* (J/g)	*T_c_-T_o_* (℃)	DP6-12 (%)	DP13-24 (%)	DP25-70 (%)	ACR	AFST
520S	76.29 ± 0.06 ^a^	79.42 ± 0.28 ^a^	83.28 ± 0.14 ^a^	13.87 ± 0.43 ^a^	6.99 ± 0.05 ^a^	25.48 ± 0.15 ^a^	54.44 ± 0.34 ^a^	22.83 ± 0.18 ^a^	0.17 ± 0.01 ^a^	H
520S*wx1*	74.70 ± 0.05 ^b^	79.32 ± 0.16 ^a^	88.27 ± 0.22 ^b^	15.17 ± 0.31 ^b^	13.57 ± 0.24 ^b^	21.68 ± 0.26 ^b^	51.04 ± 0.29 ^b^	27.39 ± 0.15 ^b^	0.14 ± 0.01 ^b^	

*T_o_*, *T_p_*, *T_c_*, Δ*H*(J/g), ACR, AFST, L and H = onset, peak, and final gelatinization temperature, gelatinization enthalpy, amylopectin chain ratio, amylopectin fine structure type, high gelatinization temperature type, and low gelatinization temperature type, respectively. Values in the same column with the different letter is significantly (*p* < 0.05). Note: Refer to Carbohydrate Polymers for the calculation method of ACR.

## Data Availability

The original contributions presented in the study are included in the article/Supplementary Material, further inquiries can be directed to the corresponding author.

## Supplementary Materials

The following supporting information can be downloaded at: https://www.mdpi.com/article/10.3390/foods14203530/s1. Figure S1. Panicle type, Grain type, Number of spikelets per panicle, Grain width, Grain length, Fertility identification. (a) Panicle type, Grain type, Number of spikelets per panicle, Grain width, Grain length of *wx* mutants and their corresponding WTs. (b) Fertility identification of *wx* mutants and their corresponding WTs. Spikelet and I_2_-KI staining of pollen of *wx* mutants and WT by stereomicroscope, temperature above 24 °C. Spikelet and I_2_-KI staining of pollen of *wx* mutants and WT by stereomicroscope, temperature under 24 °C. Figure S2. Detection of transgene DNA in *wx* lines in 520S background. Table S1. Primers used in this study. Table S2. Percentage of T0 plants with mutations in the target locus.
