# Supplementary material for: Creation of TGMS Lines of Waxy Rice with Elite Physicochemical Properties of Starch via Waxy Gene Editing
Source: Foods. 2025 Oct 16;14(20):3530. doi: 10.3390/foods14203530 (PMC12563075; doi:10.3390/foods14203530)
Supplement: Supplementary file 1 [file foods-14-03530-s001.zip › foods-3900327-supplementary Table S1.pdf]

Table S1. Primers used in this study.

Primers	Forward primer	Reverse primer
Target1-412	TGCAGAGATCTTCCACAGCA	TTGAAGACGACGACGGTCAG
Cas9-415	CACCATCTACCACCTGAGAA	CGAAGTTGCTCTTGAAGTTG
Waxy-416	GTTGGAAGCATCACGA	TAACCGGATTTGAACG
Actin	CTTCATAGGAATGGAAGCTGCGGGTA	CGACCACCTTGATCTTCATGCTGCTA
