# Supplementary material for: Creation of TGMS Lines of Waxy Rice with Elite Physicochemical Properties of Starch via Waxy Gene Editing
Source: Foods. 2025 Oct 16;14(20):3530. doi: 10.3390/foods14203530 (PMC12563075; doi:10.3390/foods14203530)
Supplement: Supplementary file 1 [file foods-14-03530-s001.zip › foods-3900327-supplementary Table S2.pdf]

Table S2. Percentage of T0 plants with mutations in the target locus.

Target site	Host cultivar	No. of plants examined	No. of plants with mutations	Mutation rate (%)	Putative homozygous mutations		Putative bi-allelic mutations		Putative heterozygous mutations	
					Number	%	Number	%	Number	%
Target 1	520S	13	12	92.3	11	84.6	0	0	0	0
