# Supplementary figures and images for: Creation of TGMS Lines of Waxy Rice with Elite Physicochemical Properties of Starch via Waxy Gene Editing
Source: Foods. 2025 Oct 16;14(20):3530. doi: 10.3390/foods14203530 (PMC12563075; doi:10.3390/foods14203530)

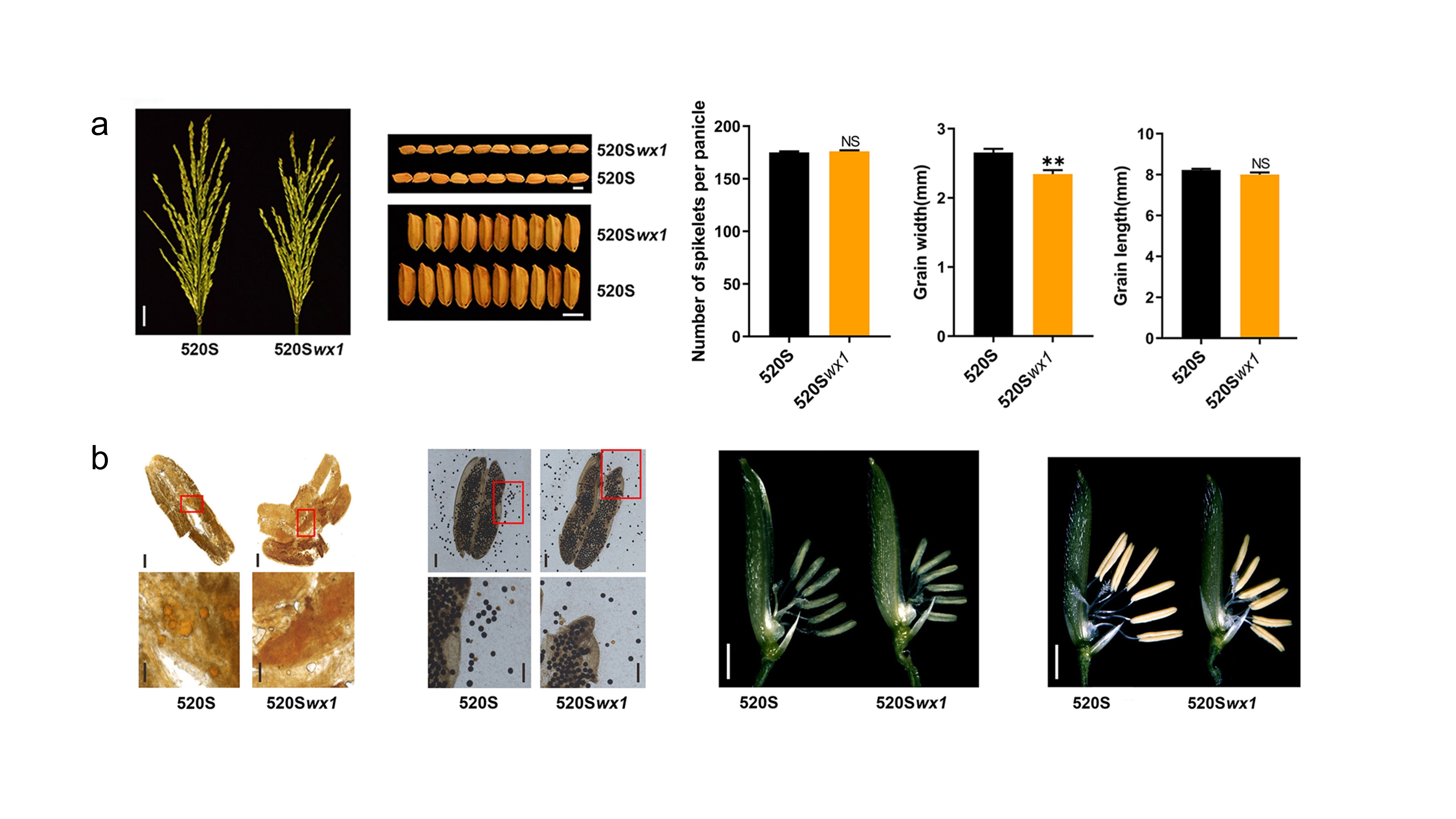

Supplement: Supplementary file 1 [file foods-14-03530-s001.zip › foods-3900327-supplementary Figure S1.jpg]

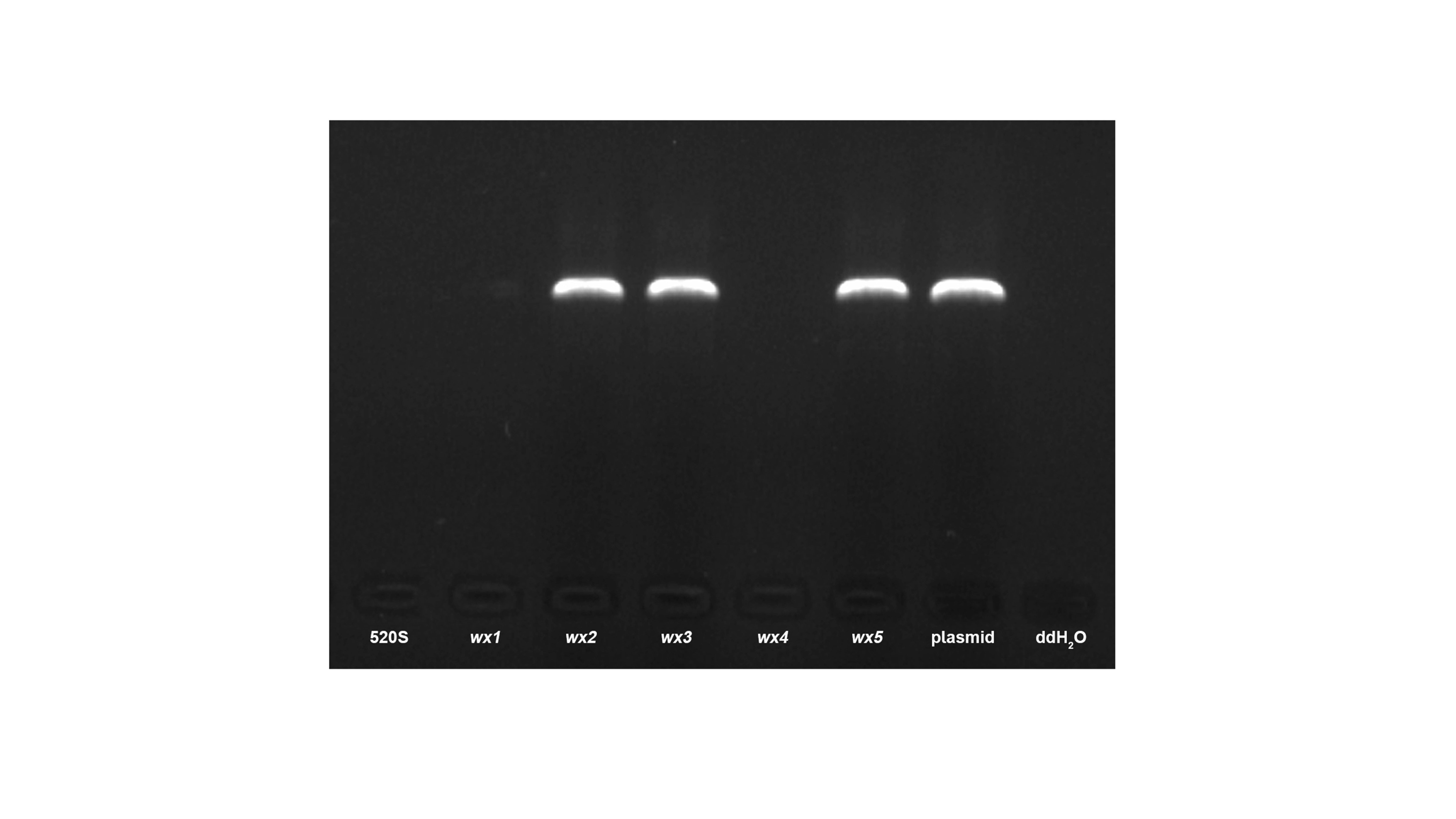

Supplement: Supplementary file 1 [file foods-14-03530-s001.zip › foods-3900327-supplementary Figure S2.jpg]
